# Supplementary material for: A Microwave-Assisted Boudouard Reaction: A Highly Effective Reduction of the Greenhouse Gas CO2 to Useful CO Feedstock with Semi-Coke
Source: Molecules. 2021 Mar 10;26(6):1507. doi: 10.3390/molecules26061507 (PMC8001657; doi:10.3390/molecules26061507)
Supplement: Supplementary file 1 [file molecules-26-01507-s001.pdf]

molecules

MDPI

Supplementary material

Article

Microwave assisted the Boudouard reaction: Highly effective reduction of the greenhouse gas CO_2 to useful CO feedstock with Semi-Coke

Huan Dai [1,2], Hong Zhao [1,*], Siyuan Chen[3] and Biao Jiang [3,*]

1 Green Chemical Engineering Research Center, Shanghai Advanced Research Institute, Chinese Academy of Sciences, Shanghai 201210, China; daihuan2018@sari.ac.cn (H.D.); zhaoh@sari.ac.cn (H.Z.)
2 University of Chinese Academy of Sciences, Beijing 100049, China; daihuan2018@sari.ac.cn
3 Shanghai Green Chemical Engineering Research Center, Shanghai Institute of Organic Chemistry, Chinese Academy of Sciences, Shanghai 200032, China; jiangb@mail.sioc.ac.cn (B.J.); chensy@sioc.ac.cn (S.C.)
* Correspondence: jiangb@sioc.ac.cn; zhaoh@sari.ac.cn

molecules

Table of contents:

--Figure S1 Schematic diagram of microwave experiment device
--Table S1 Experiment Data

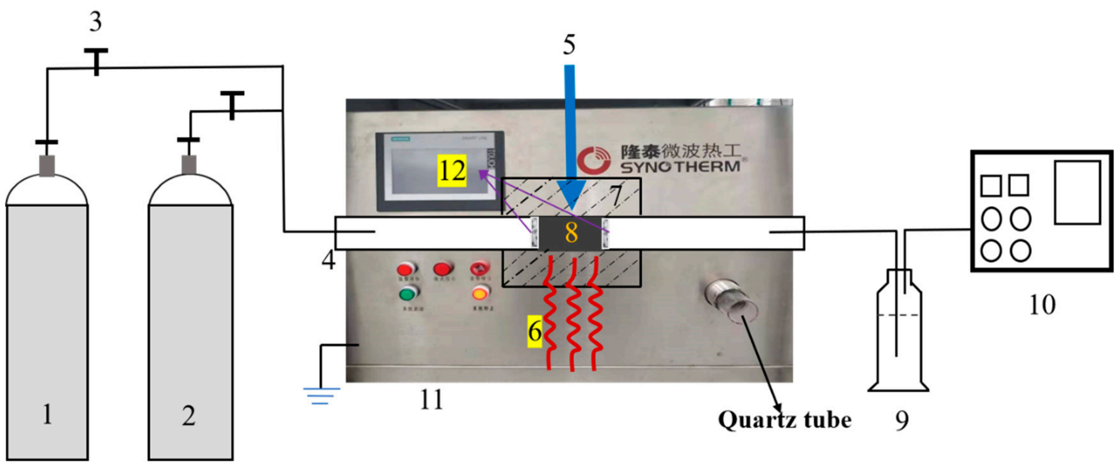

Figure S1 Schematic diagram of microwave experiment device (1. CO2 gas; 2. Ar gas; 3. Mass flow controller; 4. Quartz tube; 5. Infrared thermometer; 6. Microwave source; 7. Insulation materials; 8. Sample; 9. Filter bottle; 10. Micro GC; 11. Microwave furnace; 12. High temperature quartz fiber).

Table S1 Experiment Data

**Thermal field**	**Temperature/ °C**	**Sample/g**	**CO_2 flow /ml/min**	**Particle size/ mm**	**Catalytic/ 5wt.%**	**X_{SC}/ %**	**CO output /mmol**
Conventional heating	800	5	20	0.200-0.450	-	0.6	8.1
	850	5	20	0.200-0.450	-	1.6	15.2
	900	5	20	0.200-0.450	-	4.0	30.4
	950	5	20	0.200-0.450	-	7.4	59.1
Microwave heating	750	40	175	0.200-0.450	-	18.0	1163
	800	40	175	0.200-0.450	-	25.8	1562
	850	40	175	0.200-0.450	-	29.0	1849
	900	40	175	0.200-0.450	-	34.8	2000
	800	40	125	>0.200	-	23.1	1329
	800	40	125	0.200-0.450	-	23.7	1426
	800	40	125	0.450-0.850	-	18.7	1191
	800	40	125	0.850-1.400	-	16.2	1082
	800	40	150	0.200-0.450	-	24.9	1558
	800	40	200	0.200-0.450	-	26.5	1581
	750	40	200	0.200-0.450	-	25.6	1689
Microwave heating	750	40	200	0.200-0.450	CaO	68.2	2392
	750	40	200	0.200-0.450	$BaCO_3$	66.6	2520
